# Conjugation of a Hybrid Plasmid Encoding Hypervirulence and Carbapenem Resistance in *Klebsiella pneumoniae* of Sequence Type 592

**DOI:** 10.3389/fmicb.2022.852596

**Published:** 2022-04-25

**Authors:** Qian Zhao, Yu Feng, Zhiyong Zong

**Affiliations:** ^1^Center of Infectious Diseases, West China Hospital, Sichuan University, Chengdu, China; ^2^Center for Pathogen Research, West China Hospital, Sichuan University, Chengdu, China; ^3^Department of Infection Control, West China Hospital, Sichuan University, Chengdu, China

**Keywords:** β-lactamases, carbapenemases, resistance, virulence, conjugation, *Klebsiella pneumoniae*

## Abstract

*Klebsiella pneumoniae* simultaneously carrying genes encoding carbapenem resistance and hypervirulence causes fatal infections, representing a severe threat to human health. These carbapenem-resistant and hypervirulent *K. pneumoniae* (hvCRKP) strains are increasingly reported worldwide and have been found to belong to a variety of sequence types (STs). In this study, we report and characterized an hvCRKP strain of ST592, an uncommon ST, which caused a fatal infection in intensive care unit (ICU) in China and represents a novel type of hvCRKP. We demonstrated that this novel hvCRKP type emerged from the carbapenem-susceptible hypervirulent *K. pneumoniae* (hvKP) lineage of the K57 capsular type. K57 hvKP contains a pLVPK-like virulence plasmid and then acquired a conjugative *bla*_KPC–2_-carrying plasmid to form hvCRKP. The pLVPK-like virulence plasmid contains no complete conjugation module but was able to be transferred by fusion with the conjugative *bla*_KPC–2_-carrying plasmid during conjugation. This represents a new mechanism of simultaneous transfer genetic determinants of carbapenem resistance and virulence and highlights the undergoing expansion of hvCRKP, which requires rigorous monitoring and novel countermeasures to curb plasmid-mediated transmission.

## Introduction

Hypervirulent and carbapenem resistant *Klebsiella pneumoniae* (hvCRKP) has been increasingly reported worldwide since 2015 (Zhang et al., 2015; Russo and Marr, 2019; Wyres et al., 2020; Yang et al., 2021). It has been reported that hvCRKP strains are diverse in clonal background belonging to various sequence types (STs) such as ST11, ST14, ST23, ST25, ST36, ST65, and ST86 and carry a variety of carbapenemase-encoding genes such as *bla*_KPC–2_, *bla*_NDM–5_, and *bla*_OXA–48_ (Wyres et al., 2020; Yang et al., 2021). Such diverse clonal background of hvCRKP strains is due to two common evolutionary paths including the acquisition of virulence factors by carbapenem resistant *K. pneumoniae* (CRKP) strains or the acquisition of carbapenem resistance by hypervirulent *K. pneumoniae* (hvKP), and the former path appears to be more common than the latter (Wyres et al., 2019). Hypervirulence of hvCRKP is typically encoded by a pLVPK-like plasmid carrying multiple virulence factors including *rmpADC* and *rmpA2* (regulators of the mucoid phenotype), aerobactin-encoding *iucABCD-iutA*, and salmochelin-encoding *iroBCDN* (Russo and Marr, 2019; Yang et al., 2021). In addition, the pLVPK-like plasmid carries *ter*, a potassium tellurite resistance operon (Russo and Marr, 2019; Yang et al., 2021). Recently, hvCRKP of new clonal background is continuously emerging, while the underlying mechanism mediating the evolution of hvCRKP remains to be elucidated (Yang et al., 2021). During our clinical practice, we found a CRKP clinical isolate carrying multiple virulence factors (*rmpADC* and *rmpA2*, *iucABCD-iutA*, and *iroBCDN*) but belonging to ST592, an uncommon sequence type associated with hypervirulence. This promoted us to characterize this strain in detail and to examine the transfer potential of virulence genes from this strain.

## Materials and Methods

### The Strain and *in vitro* Susceptibility

Strain 090515 (also called WCHKP090515) was isolated from sputum of a male ICU patient with severe pneumonia and septic shock in July 2019 in West China Hospital as part of routine care. Preliminary species identification and *in vitro* antimicrobial susceptibility were performed by Vitek II (bioMérieux, Marcy-l’Étoile, France). In addition, MICs of ceftazidime-avibactam, colistin, imipenem, and meropenem were determined using the broth microdilution method and MICs of fosfomycin was determined using the agar dilution method according to the Clinical and Laboratory Standards Institute (CLSI) (CLSI, 2020). As no breakpoints for tigecycline are provided by CLSI, the breakpoint of tigecycline (0.5 mg/L) defined by EUCAST was adopted.^1^ The study was approved by the Ethical Committee of West China Hospital with inform consent being waived.

### Whole Genome Sequencing and Analysis

To obtain the complete genome sequences, 090515 and its transconjugant Tx090515vir (see below) were subjected to both short-read sequencing using a HiSeq X10 sequencer (Illumina, San Diego, CA) and long-read sequencing with a MinION sequencer (Nanopore, Oxford, United Kingdom). *De novo* hybrid assembly of both short and long reads were performed using Unicycler^2^ under the conservative mode. Sequence types were assigned by querying the multi-locus sequence typing database.^3^ Capsule types and virulence factors were investigated using Kleborate v2.0.1.^4^ Acquired antimicrobial resistance genes and plasmid replicons were identified using ResFinder and PlasmidFinder (both at),^5^ respectively. The circular plasmid map was generated using BLAST Ring Image Generator v.0.95.22 (Alikhan et al., 2011).

### Conjugation Experiments

Conjugation experiments of strain 090515 were performed using the filter-mating method as described previously (Valenzuela et al., 2007). The azide-resistant *E. coli* J53 AziR and *Klebsiella* 700603 AziR (azide-resistant variant of J53 and ATCC 700603, respectively) were used as the recipients. Potential transconjugants were selected on LB agar plates containing 2 mg/L meropenem and 150 mg/L azide for the *bla*_KPC–2_-carrying plasmid or containing 32 mg/L potassium tellurite (tellurite resistance is attributed to the presence of the *ter* operon) and 150 mg/L azide for the hypervirulence-encoding plasmid. Possible transconjugants were confirmed by amplifying *bla*_KPC–2_, *rmpA*, and *iucA* (primers listed in Table 1), respectively. Conjugation frequency was equal to the ratio of transconjugants to recipients. MICs of meropenem and potassium tellurite against the transconjugants were determined using the broth microdilution method (Yang et al., 2019; CLSI, 2020).

**TABLE 1:** Primers used in the study.

Gene/sequence	Primers	Sequence(5′– 3′)
*iucA*	iuc-F	TTCCGAGTGGTAGTGGTT
	iuc-R	GTGGCTTGAAGAGTGAGAA
*rmpA*	rmpA-F	ATTGGTTGACAGCAGGATT
	rmpA-R	GTTAGCCGTGGATAATGGT
*bla*_KPC_	KPC-F	ATGTCACTGTATCGCCGTCT
	KPC-R	TTTTCAGAGCCTTACTGCCC
*repFIB*_K_	repFIB_K__F	CTATCTGGATTACACCGAGTT
	repFIB_K___R	CCTTCTCTTCTTCCTCCTTG
*repFII_pKP91_*	repFII_pKP91__F	GTGTCCTGGCTGTATCTG
	repFII_pKP91__R	TGTTGTTCCGCATCAAGT
Fusion site 1	FS_1_F	TTTTCCTGAACACTGCGCCG
	FS_1_R	GTGAAATAGAACAGGTGACC
Fusion site 2	FS_2_F	CATTTTCGGTCCCATTCTGGC
	FS_2_R	GCTACAGGCAGTCTTCGGCG

*All primers are self-designed. However, the primers for bla_KPC–2_ are identical to those used in a study (Carattoli et al., 2021), which is likely a coincidence. The primers for iucA have been published by us in another study (Zhao et al., 2022).*

Conjugation assay was also performed to examine whether the hybrid plasmid identified in this study (see below for details) could be transferred to clinical CRKP strains. Tx090515vir, the J53 transconjugant containing the hybrid plasmid obtained from the above conjugation experiment and an amikacin-resistant ST11 K64 CRKP clinical strain 115112 were used as the donor and the recipient, respectively. The chromogenic agar medium containing 32 mg/L potassium tellurite and 64 mg/L amikacin was used to select the probable transconjugants. The replicon genes of IncFIB_K_ (*repFIB*_K_) and IncFII_pKP91_ (*repFII_pKP91_*) and the sequence crossing the two fusion sites (assigned FS_1 and FS_2 here) of the hybrid plasmid pKPC-2_Vir (see below for details) were amplified (primers listed in Table 1) to further verify the conjugation.

### Virulence Assay Using *Galleria mellonella* Larvae and Murine Intraperitoneal Infection Model

The virulence of strain 090515 was determined using *Galleria mellonella* (wax moth) larvae as described previously (Peleg et al., 2009). Strain 090276 (ST11 K64/*wzi*_64 hvCRKP) and 090249 (ST23 K1/*wzi*_1 hvCRKP) were used as the hypervirulence control, while 090267 (ST11 K64/*wzi_*64 non-hypervirulent CRKP, Table 2) was the low virulence control. Briefly, 16 larvae of 250–350 mg in weight (Huiyude; Tianjin, China) were randomly assigned to each group. Strains were washed using phosphate-buffered saline (PBS; Beyotime, Shanghai, China) and adjusted to 0.5 McFarland (around 10^8^ colony forming units (CFU)/mL). Each larva was injected with 10 μL bacterial dilution (10^5^, 10^6^, and 10^7^ CFU, respectively) using a microsyringe (Gaoge, Shanghai, China). Larvae injected with 10 μL PBS alone were used as negative control. Under incubation at 37°C, the survival of larvae was recorded at 12 and 24 h and the virulence assay was performed in triplicate. The result was described as mean ± standard deviation (SD). An unpaired two-sided Student’s *t*-test was performed to analyze the statistical difference using SPSS 26.0 (IBM Analytics; Armonk, NY).

**TABLE 2:** MIC (mg/L) of antimicrobial agents and potassium tellurite for strains in this study.

Strain	Description	IMP	MER	TIG	COL	CAZ- AVI	AMK	CIP	CTX	FOS	PT
090515	ST592 K57 hvCRKP	32	16	2	1	1	4	16	≥ 64	32	≥64
090267	ST11 K64 CRKP, control for virulence assay	64	32	1	0.5	4	≥ 64	16	≥ 64	512	< 1
090276	ST11 K64 hvCRKP, control for virulence assay	64	32	1	0.5	4	4	16	≥ 64	64	≥64
090249	ST23 K1 hvCRKP, control for virulence assay	64	32	16	1	0.5	≥ 64	16	≥ 64	32	≥64
J53 AziR	*E. coli*, for conjugation	0.13	0.06	< 0.5	<0.5	< 0.5	<2	< 0.25	<1	8	< 1
700603 AziR	*Klebsiella*, for conjugation	0.13	0.06	< 0.5	<0.5	< 0.5	4	<0.25	< 1	> 512	4
115112	ST11 K64 CRKP, for conjugation	512	256	2	1	1	≥ 64	16	≥ 64	512	< 1
Tx090515KPC-2	700603 AziR transconjugant with pKPC-2_090515	4	8	< 0.5	<0.5	< 0.5	<2	16	≥ 64	512	4
Tx090515vir	J53 AziR transconjugant with pKPC-2_Vir	2	2	< 0.5	<0.5	< 0.5	4	16	≥ 64	2	≥64
Tx01	A derivate of Tx090515vir losing pKPC-2_Vir in sub-cultivating	0.13	0.06	< 0.5	<0.5	< 0.5	4	<0.25	< 1	8	<1
Tx115112	115112 Transconjugant with pKPC-2_Vir	512	128	2	1	1	≥ 64	16	≥ 64	512	≥ 64

*IMP, imipenem; MER, meropenem; TIG, tigecycline; COL, colistin; CAZ-AVI, ceftazidime-avibactam; AMK, amikacin; CIP, ciprofloxacin; CTX, cefotaxime; FOS, Fosfomycin; PT, potassium tellurite.*

The virulence of the strains was further examined using murine intraperitoneal infection model as described previously (Russo et al., 2021). Six-week-old wild-type C57BL/6J mice (weight, 18–21 g) were obtained from ENSIWEIER (Chengdu, China) and were maintained in a standard animal facility at the Laboratory Animal Center of Sichuan University. Mice were feed for 5 days and then randomly assigned to each group. Mice were subsequently injected with 100 μL bacterial dilution (10^7^CFU) using 1 ml insulin syringes (Shifeng, Chengdu, China). The survival of mice was recorded every 24 h for 3 days and a survival curve was plotted using Prism 8.0.2 (GraphPad Software, Inc., San Diego, CA). Statistical analysis was performed using the log-rank (Mantel–Cox) test. The animal experiments were approved by the Ethics Committee for Laboratory Animals of West China Hospital.

In addition, the virulence of the recipient strains 115112 and J53 AziR as well as the transconjugants Tx090515Vir and Tx115112 was determined using both larvae and mice as described above.

### Hypermucoviscous Assay and Quantification of Capsule

Hypermucoviscous assay was performed using the string test by stretching the bacterial colonies on sheep blood agar plates using an inoculation loop (Shon et al., 2013). The capsule production was determined by quantifying glucuronic acid content using a colorimetric assay as described previously (Palacios et al., 2018). The experiments were repeated in triplicate and glucuronic acid content was presented as mean ± SD. The statistical analysis was performed with an unpaired two-sided Student’s *t*-test using SPSS 26.0.

### Fitness Assay

Fitness conferred by the hybrid plasmid, pKPC-2_Vir, was determined using both the growth curve assay and competition experiments. The growth curve assay was performed as described previously (Zhang et al., 2020). The parental recipient strains J53 AziR and 115112, the corresponding plasmid-carrying transconjugant strain Tx090515vir and Tx115112, and the plasmid-losing transconjugant strain Tx01 during sub-cultivating were cultured in LB broth overnight and diluted to 0 of OD_600 nm_ and then grown at 37°C with rotation (180 r.p.m). The cultured cell density was determined every 0.5 h by measuring the OD_600 nm_ for 9 h till the plateau phase.

Competition experiments between Tx115112 (carrying pKPC-2_Vir) and its parental strain 115112 were performed as described previously (San Millan et al., 2010). Briefly, Tx115112 (100 μL, 10^6^ CFU) and 115112 (100 μL, 10^6^ CFU) were added in 10 ml LB broth without potassium tellurite, respectively, and were then incubated at 37°C at 180 r.p.m for 24 h. Before and after the incubation, 100 μL aliquots of the cultures were retrieved and streaked on LB agar plates with and without 32 mg/L potassium tellurite plus 2 mg/L meropenem to represent the bacterial load (CFU) at the start time point (t0) and that after 24 h incubation (t24). The competition index (CI) was used to describe the fitness cost, which is equal to the ratio of the bacterial CFU for the transconjugant and the recipient strain at t24 divided by the counterpart at t0 (Gutierrez et al., 2012).

### Plasmid Stability Assay

The plasmid stability was performed as described previously (Dionisio et al., 2005; Ma et al., 2018). Briefly, transconjugant strains Tx090515vir and Tx115112 were cultured in LB broth at 37°C with rotation (180 r.p.m) for 14 days and the medium was refreshed every 12 h. The sub-cultivating generations were calculated according to the formula N_t_ = N_0_ × 2*^nt^* with N_t_ representing the colony count at time *t*, N_0_ representing the colony count at the beginning, and nt representing bacterial cell division times. The replicon genes, *rmpA*, *iucA*, and *bla*_KPC–2_ were amplified using DNA prepared from the broth in sub-cultivating to verify the presence of the hybrid plasmid pKPC-2_Vir, and the sequences crossing the fusion site were amplified to assure the integrity of the hybrid plasmid. In addition, 100 μL of cultures (the concentration of 10^3^ CFU) before or after sub-cultivating were streaked onto a LB agar plate with 32 mg/L potassium tellurite and 2 mg/L meropenem, one with 32 mg/L potassium tellurite, one with 2 mg/L meropenem and one without meropenem nor potassium tellurite, respectively. The plasmid stability was calculated according to the formula log_10_(*Ng*)/log_10_(*Nw*), where *Ng* represents the colony count on the medium with meropenem, potassium tellurite, or both, while *Nw* represents that on the medium without meropenem nor potassium tellurite.

### Identification of Homologous Regions

The hybrid plasmid pKPC-2_Vir was compared to its parental plasmids carrying *bla*_KPC–2_ or encoding hypervirulence by BLAST^6^ with default search parameters (general parameters, scoring parameters, and filters and masking) and manually checking to identify the sites of plasmid fusion. Surrounding 500-bp sequences at either side of the 12-bp identical sequence at the fusion sites (see below for details) on the *bla*_KPC–2_-carrying plasmid were compared with the counterparts on the hypervirulence-encoding plasmid by BLAST with a word size of 7 and otherwise default search parameters to identify homologous regions across the fusion sites.

## Results

### The Strain Represents a New Type of Hypervirulent and Carbapenem Resistant *Klebsiella pneumoniae*

The complete genome sequence of 090515 was obtained with *de novo* hybrid assembly of both short and long reads. 090515 belongs to ST592 (*gapA-infB-mdh-pgi-phoE-rpoB-tonB* allele no. 2-3-6-1-9-4-13) and K57 (*wzi*_206) capsular type. 090515 has a 5.06-Mb chromosome, a 133-kb *bla*_KPC–2_-carrying plasmid (assigned pKPC-2_090515 here) and a 218-kb plasmid with multiple virulence factors including *rmpADC* and *rmpA2*, *iucABCD-iutA*, and *iroBCDN* (assigned pVir_090515 here; Table 3). This indicates that the strain encodes hypervirulence and carbapenem resistance simultaneously. As ST592 hvCRKP has not been reported before, strain 090515 represents a new type of hvCRKP.

**TABLE 3:** The complete genome, antimicrobial resistance genes, and virulence factors of strain 090515.

090515	Accession no.	Size (bp)	Replicon type	Virulence gene	Resistance gene
Chromosome	CP073287	5,060,265	–	*mrkABCDFHIJ*	*bla*_SHV–26_, *fosA5*, *oqxA9*, *oqxB12*
pKPC-2_090515	CP073288	133,256	IncFII_*Yp*_, IncFII_pKP91_, IncR	–	*dfrA14*, *qnrB1*, *bla*_KPC–2_
pVir_090515	CP073289	218,309	IncFIB_K_, IncHI1B_pNDM–Mar_	*iucABCD-iutA*, *iroBCDN*, *rmpADC*, *rmpA2*	–

**FIGURE 1:**
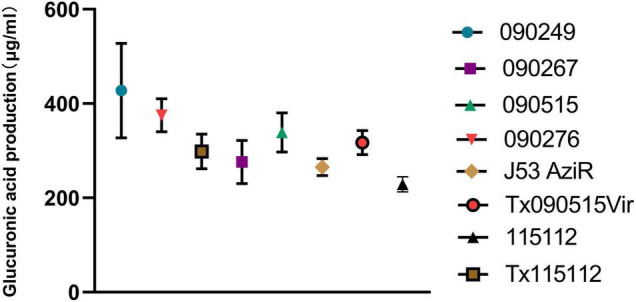
Glucuronic acid production of different bacterial strains. 090515 (ST592 K57 hvCRKP), Tx090515vir (*E. coli* J53 AziR transconjugant containing pKPC-2_Vir), and Tx115112 (transconjugant of 115112 containing pKPC-2_Vir) were assayed. 090276 (ST11 K64 hvCRKP) and 090249 (ST23 K1 hvCRKP) were used for positive hypermucoviscous controls, while 090267 (ST11 K64, non-hypervirulent CRKP), J53 AziR (*E. coli*, the recipient strain of pKPC-2_Vir) and 115112 (ST11 K64 CRKP, the recipient strain of pKPC-2_Vir) were used for non-mucoviscous controls.

Strain 090515 exhibited resistance to imipenem (MIC, 16 mg/L), meropenem (MIC, 32 mg/L), and tigecycline (MIC, 2 mg/L), but was susceptible to ceftazidime-avibactam (MIC, 0.25 mg/L), colistin (MIC, 1 mg/L) and fosfomycin (MIC, 32 mg/L) (Table 2). This confirms carbapenem resistance in the strain. In addition, this strain was also resistant to aztreonam (MIC, 16 mg/L) and piperacillin-tazobactam (MIC, ≥ 128/4 mg/L) but was susceptible to amikacin (MIC, ≤ 2 mg/L), ciprofloxacin (MIC, 1 mg/L), gentamicin (MIC, ≤ 1 mg/L), and trimethoprim-sulfamethoxazole (MIC, 2/38 mg/L) as determined by Vitek II. 090515 was not hypermucoviscous in the string test. The glucuronic acid quantification assay showed that the capsule production of strain 090515 was similar to that of 090276 (a ST11 K64 hvCRKP, 338.69 ± 41.48 vs. 375.12 ± 34.99 μg/ml, *p* = 0.31) but appeared to be lower (not statistically significant, 338.69 ± 41.48 vs. 427.50 ± 100.21 μg/ml, *p* = 0.23) than that of 090249 (a ST23 K1 hvCRKP, Figure 1). The patient received polymyxin B and tigecycline in combination (ceftazidime-avibactam was not available at that time). Unfortunately, his conditions deteriorated, and he died 30 days after ICU admission.

**FIGURE 2:**
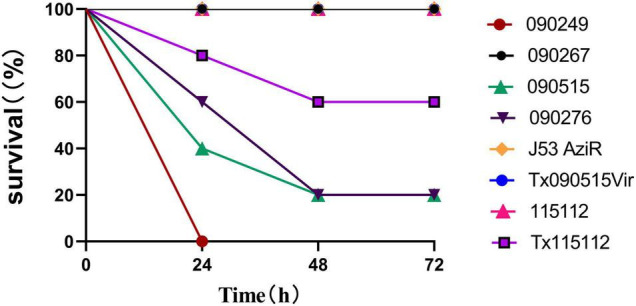
Virulence potential of different bacterial strains as depicted in the murine intraperitoneal infection model with an inoculum of 10^7^ CFU. Percentages (%) of mice (*n* = 5 for each strain) survived after infected with the corresponding strain at an inoculum of 10^7^ at different time points (h) are shown. 090515 (ST592 K57 hvCRKP), Tx090515vir (*E. coli* J53 AziR transconjugant containing pKPC-2_Vir), Tx115112 (CRKP 115112 transconjugant containing pKPC-2_Vir) were assayed. 090276 (ST11 K64 hvCRKP) and 090249 (ST23 K1 hvCRKP) were used for positive (hypervirulent) controls, while 090267 (ST11 K64, non-hypervirulent CRKP), J53 AziR (*E. coli*, the recipient strain of pKPC-2_Vir) and 115112 (ST11 K64 CRKP, the recipient strain of pKPC-2_Vir) were used for low virulence controls. PBS was used for the negative control.

In the *G. mellonella* larvae infection model, the survival rate of larvae infected with 090515 was similar to that of larvae infected with the hypervirulent 090276 and 090249 and was significantly lower than that of those with the non-hypervirulent 090267 (Supplementary Table 1). In the murine intraperitoneal infection model, strain 090515 led to 60% death of infected mice at 24 h, while 090276 and 090249 resulted in 40 and 100% death. In contrast, none of mice infected with the low virulent control strain 090267 died at 72 h (*P* < 0.0001, Figure 2). Virulence assays using larvae and mice confirmed the hypervirulence nature of strain 090515.

### *bla*_KPC–2_ Was Located in a Type II Non-tn*4401* Element (NTE_KPC_-II) on a Self-Transmissible Plasmid

The plasmid pKPC-2_090515 contains three replicons (an IncFII_*Y*p_, an IncFII_pKP91_, and an IncR). pKPC-2_090515 could be transferred to *Klebsiella* 700603 AziR at a frequency of 4.6 × 10^–5^ (the ratio of transconjugants to recipients) but failed to be transferred to J53 AziR.

pKPC-2_090515 had the highest nucleotide identity (82% coverage and maximum 99.86% identity, Supplementary Figure 1) with pOXA1-191663 (GenBank accession no. CP080359), an IncFII_K_ and IncR plasmid carrying no *bla*_KPC–2_ from a ST16 *K. pneumoniae* in China. On pKPC-2_090515, *bla*_KPC–2_ was located into a variant of NTE_KPC_-II element (NTE stands for non-Tn*4401* element) containing a truncated *bla*_TEM–1_ (encoding a narrow-spectrum β-lactamase). This NTE_KPC_-II element was flanked by two copies of IS*26*, which could form a composite transposon mediating the mobilization of this NTE_KPC_-II element (Supplementary Figure 1).

### The Virulence-Encoding Plasmid Contains No Conjugation Module but Could Be Transferred by Fusion With the *bla*_KPC–2_-Carrying Plasmid During Conjugation

The plasmid pVir_090515 contains an IncFIB_K_ and an IncHI1B_pNDM–Mar_ replicon, *ter*, and multiple virulence factors including *rmpADC* and *rmpA2*, *iucABCD-iutA*, and *iroBCDN*. pVir_090515 is highly similar with the well-characterized virulence plasmid pLVPK (GenBank accession no. AY378100) with 96% coverage and 99.97% nucleotide identity (Supplementary Figure 2). The conjugative module comprising *tra* genes is completely absent from pVir_090515 (Supplementary Figure 3) as identified by aligning with plasmid pHK23a (GenBank accession no. JQ432559) with a complete conjugative module (Ho et al., 2013). However, we obtained J53 AziR transconjugants (assigned Tx090515vir) able to grow in the presence of 32 mg/L potassium tellurite at a frequency of 7.86 × 10^–7^, while no transconjugants of 700603 AziR were obtained. This suggests that pVir_090515 was conjugated to *E. coli* J53 AziR but was not to *Klebsiella* 700603 AziR or was at a low conjugation frequency beyond the limit of detection (10^–8^) of this study. In addition, the plasmid pKPC-2_Vir could be transferred to 115112, a ST11 K64 CRKP clinical strain with a conjugation efficiency of 1.29 × 10^–6^ using the transconjugant Tx090515Vir as the donor strain.

**FIGURE 3:**
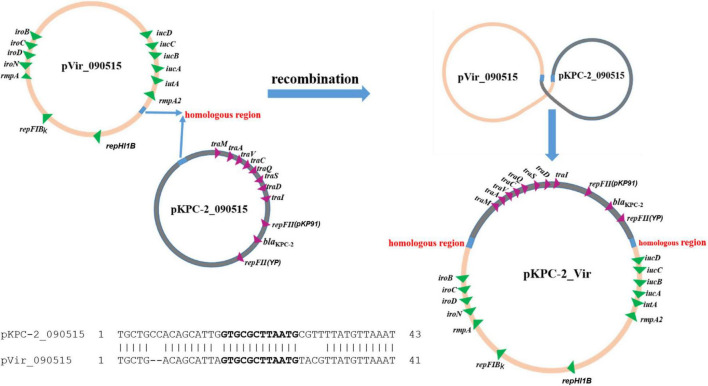
Proposed scheme for fusion of two plasmids to form pKPC-2_Vir by recombination. The two plasmids, pKPC-2_090515 and pVir_090515, had a 12-bp identical sequence (GTGCGCTTAATG, in bold) within a 43 bp homologous region (shown in cyan; 41 bp in pVir_090515) with 84% nucleotide identity (36/43 bp). Alignment of the homologous regions between pKPC-2_090515 and pVir-2_090515 is shown. Recombination between the two homologous regions could result in fusion of the two plasmids form a single large hybrid plasmid (pKPC-2_Vir) encoding both carbapenem resistance and virulence. Genes encoding antimicrobial resistance, virulence, plasmid replicons, and conjugation are shown.

To investigate how pVir_090515 was conjugated, we also obtained the complete genome sequence of the transconjugant Tx090515vir. Tx090515vir contains a single plasmid of 351-Kb in size, which was a co-integrate of pKPC-2_090515 and pVir_090515 (Supplementary Figure 4). Further sequence analysis showed that a 12-bp sequence (GTGCGCTTAATG) was present on both pVir_090515 (in a gene encoding a GNAT family N-acetyltransferase) and pKPC-2_090515 (in a spacer region). The homologous region containing the 12-bp identical sequence between the two plasmids extends to 43 bp (41 bp in pVir_090515) with 84% nucleotide identity (36/43 bp; the alignment is shown in Figure 3). The fusion of the two plasmids was due to recombination between the two homologous regions containing the 12-bp identical sequences (Figure 3). The fusion generated a novel conjugative hybrid plasmid (assigned pKPC-2_Vir) encoding both carbapenem resistance and hypervirulence (Figure 3 and Supplementary Figure 4).

To investigate whether the acquisition of pKPC-2_Vir could significantly increase the capsular production and enhance the virulence level, the glucuronic acid quantification assay and virulence assay were performed. The acquisition of the hybrid plasmid pKPC-2_Vir promoted the capsular production of the original recipient strain J53 AziR (317.26 ± 25.46 vs. 265.36 ± 18.03 μg/ml, *p* = 0.045) and 115112 (298.45 ± 36.86 vs. 228.69 ± 15.85 μg/ml, *p* = 0.039) (Figure 1). In the murine intraperitoneal infection model, though the virulence of the transconjugant Tx090515Vir was similar to the original recipient strain J53 AziR, the survival rate of mice infected with the transconjugant Tx115112 was significantly lower than that of the recipient strain 115112 (60 vs. 100%, *p* = 0.019, Figure 2). The above findings suggest that the hybrid plasmid pKPC-2_Vir enhanced virulence of 115112.

### The Fused Plasmid Was Stably Maintained in *Klebsiella pneumoniae*

After sub-cultivating Tx090515vir and Tx115112 for 14 days (about 973 and 704 generations, respectively) without antimicrobial agents and potassium tellurite, the replicon genes as well as the sequences crossing fusion sites were amplified successfully in most (98%) colonies of Tx115112 (*K. pneumoniae*) grew on a LB agar plate with 32 mg/L potassium tellurite and 2 mg/L meropenem. A gel electrophoresis image of PCR amplifying fusion sites is shown in Supplementary Figure 5. In contrast, no colonies able to grow on agar plates containing 32 mg/L potassium tellurite or 2 mg/L meropenem or both were recovered on day 9, suggesting that pKPC-2_Vir failed to stably maintain in Tx090515vir (*E. coli*). We therefore obtained a strain (assigned Tx01) that derived from Tx090515vir and lost pKPC-2_Vir during sub-cultivating.

**FIGURE 4:**
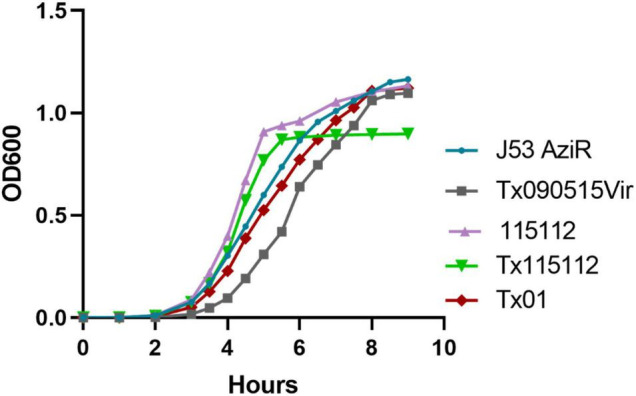
The fitness conferred by plasmid pKPC-2_Vir. Tx090515vir was J53 AziR transconjugant containing pKPC-2_Vir and Tx01 was a derivate of Tx090515vir with the loss of pKPC-2_Vir during sub-cultivating. 115112 was a ST11 K64 CRKP clinical strain and Tx115112 was a transconjugant containing pKPC-2_Vir of 115112. Bacterial growth measured by OD_600 nm_ at different time points (h) are displayed. Compared with J53 AziR, Tx090515vir had lower growth, while Tx01 displayed a similar growth rate. Compared with 115112, Tx115112 exhibited decreased growth.

Fitness conferred by the hybrid plasmid, pKPC-2_Vir, was determined. In the growth curve assay, both Tx090515vir and Tx115112 displayed decreased growth compared to its parental strain J53 AziR and 115112, respectively (Figure 4). In contrast, after the loss of pKPC-2_Vir, the growth rate of the corresponding strain Tx01 restored to that of the parental strain J53 AziR (Figure 4). The fitness was also verified through competition experiment, which showed pKPC-2_Vir conferring fitness cost to 115112 with a CI at 0.45 ± 0.06 (mean ± SD). This suggests that the large plasmid pKPC-2_Vir confers fitness cost to the host strain.

## Discussion

In this study, we report and characterized an hvCRKP strain of ST592, which is an uncommon ST associated with hypervirulence and carbapenem resistance. This highlights the diverse clonal background of hvCRKP. K57 is one of the several well-known hvKP capsular types including K1, K2, K5, K20, K54, and K57 (Fung et al., 2000; Yu et al., 2007; Russo and Marr, 2019). This suggests that strain 090515 emerged as hvCRKP by acquisition of carbapenem resistance. In consistence, the *bla*_KPC–2_-carrying plasmid of 090515 was conjugative. K57 *K. pneumoniae* strains can be assigned to a few STs including ST1, ST11, ST161, ST218, ST268, ST412, and ST592 (Ma et al., 2020; Wei et al., 2021), among which ST412 is dominant (Wei et al., 2021). ST592 K57 strains have sporadically been reported in China (Lin et al., 2015; Guo et al., 2017) and southeast Asia (Mobasseri et al., 2020; Wyres et al., 2020) but none of these strains were resistant to carbapenems. Therefore, ST592 hvCRKP has not been reported before.

hvCRKP has been increasingly reported recently, in particular in China (Liu et al., 2020; Yang et al., 2021). Typically, the carbapenemase gene and virulence genes are carried by separated plasmids in hvCRKP strains (Yang et al., 2021) like 090515 seen in this study. In hvKP, the pLVPK-like virulence plasmids had long been thought to be non-conjugative due to the lack of a complete conjugative module comprising *tra* genes (Yang et al., 2021). However, several recent studies have found that the pLVPK-like virulence plasmids can become mobilizable via two major approaches (Yang et al., 2019; Xie et al., 2020; Xu et al., 2021). In the presence of a helper conjugative plasmid (Xu et al., 2021), the pLVPK-like virulence plasmid may keep as the original single plasmid to transfer alone at a very low frequency of 10^–9^ (the ratio of transconjugants to recipients) or co-transfer with the helper plasmid at a 10^–6^ to 10^–7^ frequency (Xu et al., 2021). Alternatively, the pLVPK-like virulence plasmid may be fused with a conjugative plasmid via homologous recombination (Yang et al., 2019; Xie et al., 2020) or strand exchange (Xu et al., 2021) to transfer at variable (10^–2^ to 10^–7^) frequencies (Yang et al., 2019; Xie et al., 2020). However, these studies have not demonstrated the simultaneous plasmid-mediate transfer of carbapenem resistance and virulence genes. In addition, a hybrid plasmid, pCRHV-C2244, fused by a pLVPK-like virulence plasmid and an IncFII/IncR *bla*_KPC–2_-carrying plasmid due to IS*26*-mediated transposition, has been found in a ST11 K64 hvCRKP strain (Jin et al., 2021). pCRHV-C2244 was not conjugative on its own due to lacking several *tra* genes but could be transferred to *K. pneumoniae* ATCC 13883 at a low frequency of 10^–10^ in the presence of a helper conjugative plasmid from *E. coli*. Unfortunately, it remains unknown whether pCRHV-C2244 transferred as a single plasmid or as a fused one with the helper plasmid (Jin et al., 2021). Very recently, it has been found that in a ST86 hvCRKP, a non-conjugative pLVPK-like plasmid could fuse with a conjugative *bla*_KPC–2_-carrying plasmid during conjugation via homologous recombination of a 275-bp short region and therefore realized the conjugative transfer of both plasmids as a single entity (Xie et al., 2021). In literature, it has been demonstrated that recombination can occurred between very short (<100 bp) sequence of homology (Shen and Huang, 1986; Kotani and Kmiec, 1994; Yu et al., 2000; Ira and Haber, 2002; Sagi et al., 2006; Jang et al., 2018; Lu et al., 2019) and for *E. coli*, as little as 23 bp of sequence homology are adequate for efficient recombination (Shen and Huang, 1986). In the present study, we observed that the pLVPK-like virulence plasmid can be fused with a large conjugative *bla*_KPC–2_-carrying IncFII plasmid during the conjugation via recombination. We found that such plasmid fusion-mediating recombination was likely to occur between homologous regions as short as only 43 bp. The simultaneous transfer of both genetic determinants of carbapenem resistance and virulence in a single event may facilitate more hvKP or CRKP strains to become hvCRKP, imposing great challenges for clinical management, infection control, and the combat against antimicrobial resistance.

Interestingly, in the present study the virulence-encoding plasmid could be directly transferred from the original hvCRKP strain to an *E. coli* strain but not to a *Klebsiella* strain. In contrast, when using the *E. coli* transconjugant as the donor, this plasmid could be conjugated to a *Klebsiella* strain. This discrepancy has been reported before (Xu et al., 2021) but the exact reasons remain unclear. Previously studies have suggested that the extracellular polysaccharides could serve as a barrier for plasmid transfer between bacterial strains (Anthony et al., 1994; Perez-Mendoza and de la Cruz, 2009; Wyres et al., 2019). It is possible that the extracellular polysaccharides of the two *Klebsiella*s strains (the donor and the recipient) obstruct the transfer of the large (351 kb in this case) fused plasmid. This warrants further studies.

The fused plasmid identified in the present study stably maintained in *Klebsiella*, which is consistent with previous reports about pLVPK-like plasmids (Zhang et al., 2020; Jin et al., 2021), but was prone to be lost in *E. coli*. This suggests that *E. coli* serves as an intermediate vehicle for the transfer of pLVPK-like plasmids rather than an ideal long-term host. The interaction between hypervirulence-encoding plasmids and host strains of different species needs further investigation to identify genetic factors for the discrepancy in plasmid maintenance between *K. pneumoniae* and *E. coli* as the host strain. Although previous studies have found that pLVPK-like plasmids (about 220 kb in size) seldom confer significant fitness costs for *K. pneumoniae* (Zhang et al., 2020; Jin et al., 2021), a 265-kb hybrid plasmid encoding hypervirulence causes fitness costs in different *K. pneumoniae* strains (Xie et al., 2020). The fused plasmid identified here also conferred fitness costs to both *E. coli* and *K. pneumoniae* strains. The exact reasons incurring fitness costs remain unclear but could be due to its larger size and the co-existence of five replicons due to fusion of two plasmids. This also warrants further exploration.

## Conclusion

In conclusion, we report an hvCRKP *strain* of ST592 for the first time, highlighting the continuous emergence of such a “superbug” equipped by both key antimicrobial resistance and enhanced virulence. This hvCRKP strain emerged from a K57 hvKP strain by acquiring a *bla*_KPC–2_-carrying conjugative plasmid. The acquisition of this conjugative plasmid endows the non-conjugative pLVPK-like virulence-encoding plasmid in the same strain to be mobilizable by fusion during conjugation. This represents a new event to simultaneously transfer genes encoding carbapenem resistance and virulence in a single event and may promote further emergence of hvCRKP, highlighting the urgent need of novel approaches to address plasmid-mediated transfer of key genetic determinants.

## Accession numbers

Complete sequences of the chromosome of strain 090515, plasmid pKPC_090515, pVir_090515, and pKPC-2_Vir have been deposited in the GenBank databases under accession numbers CP073287, CP073288, CP073289, and CP084027.

## Data Availability Statement

The datasets presented in this study can be found in online repositories. The names of the repository/repositories and accession number(s) can be found below: https://www.ncbi.nlm.nih.gov/genbank/, CP073287; https://www.ncbi.nlm.nih.gov/genbank/, CP073288; https://www.ncbi.nlm.nih.gov/genbank/, CP073289; https://www.ncbi.nlm.nih.gov/genbank/, CP084027.

## Author Contributions

ZZ conceived and designed the study. QZ performed the experiments. QZ, YF, and ZZ analyzed the data. QZ and ZZ wrote the manuscript. All authors have read and approved the manuscript.

## Conflict of Interest

The authors declare that the research was conducted in the absence of any commercial or financial relationships that could be construed as a potential conflict of interest.

## Publisher’s Note

All claims expressed in this article are solely those of the authors and do not necessarily represent those of their affiliated organizations, or those of the publisher, the editors and the reviewers. Any product that may be evaluated in this article, or claim that may be made by its manufacturer, is not guaranteed or endorsed by the publisher.

## Footnotes

1
http://www.eucast.org/

2
https://github.com/rrwick/Unicycler

3
https://bigsdb.pasteur.fr/klebsiella/

4
https://github.com/katholt/Kleborate

5
http://genomicepidemiology.org/

6
https://blast.ncbi.nlm.nih.gov/Blast.cgi
