# Supplementary material for: Conjugation of a Hybrid Plasmid Encoding Hypervirulence and Carbapenem Resistance in Klebsiella pneumoniae of Sequence Type 592
Source: Front Microbiol. 2022 Apr 25;13:852596. doi: 10.3389/fmicb.2022.852596 (PMC9085563; doi:10.3389/fmicb.2022.852596)
Supplement: Supplementary file 1 [file Data_Sheet_1.docx]

**Supplementary files**

**Fig. S1.** Alignment of pKPC-2_090515 and pOXA1-191663. (a). Alignment of pKPC-2_090515 and pOXA1-191663. pKPC-2_090515 had the highest nucleotide identity (82% coverage and maximum 99.86% identity (GenBank accession no. CP080359). (b) The genetic context of a variant of the type II non-Tn*4401* (NTE_KPC_-II) element on pKPC-2_090515*.*


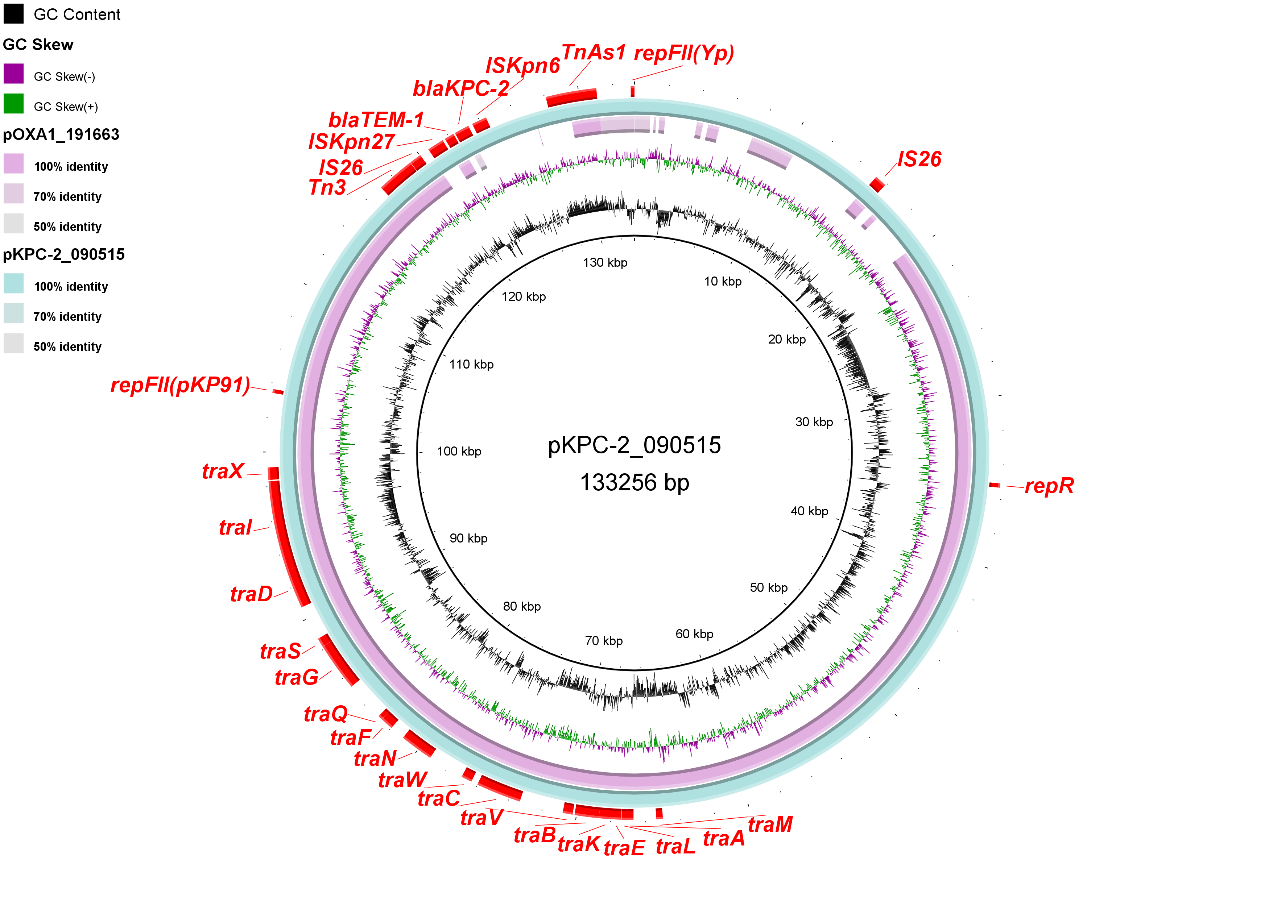


(a)


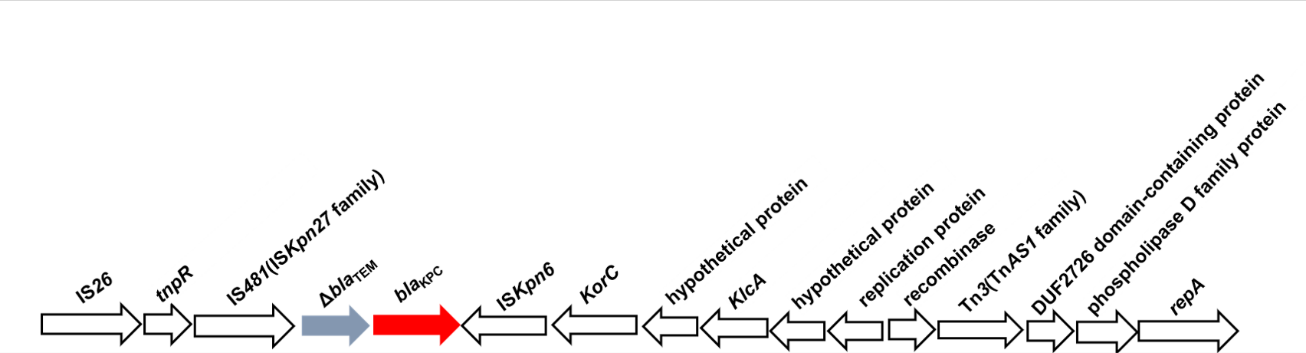


(b)

**Fig. S2.** Alignment of pVir_090515 with pLVPK. pLVPK (GenBank accession no. AY378100) is a well-characterized plasmid encoding virulence. Genes encoding virulence and plasmid replicons are shown.


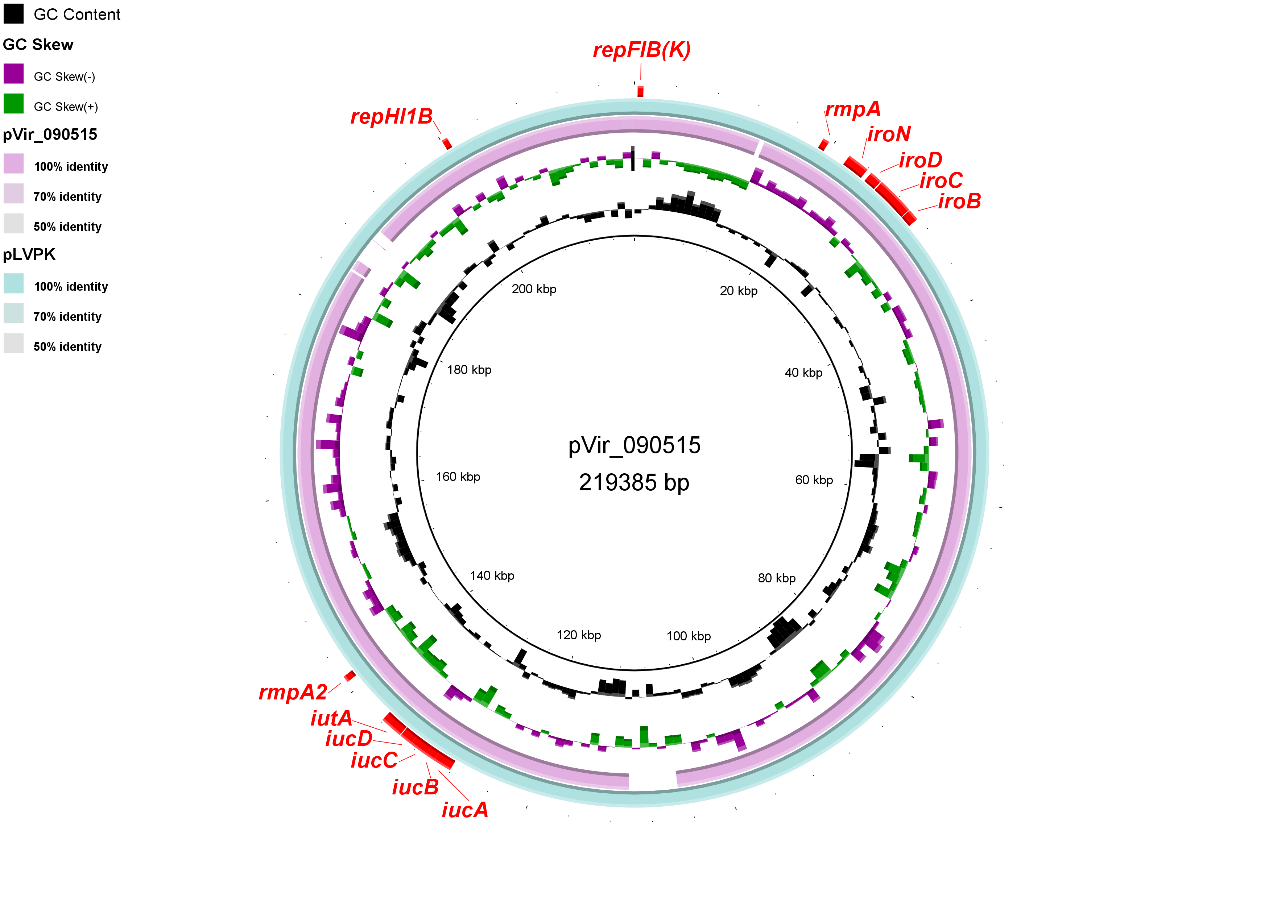


**Fig. S3. Alignment of pVir_090515 with pHK23a.** pHK23a (GenBank accession no. JQ432559) has a complete conjugative module comprising multiple *tra* genes, which is completely absent from pVir_090515.


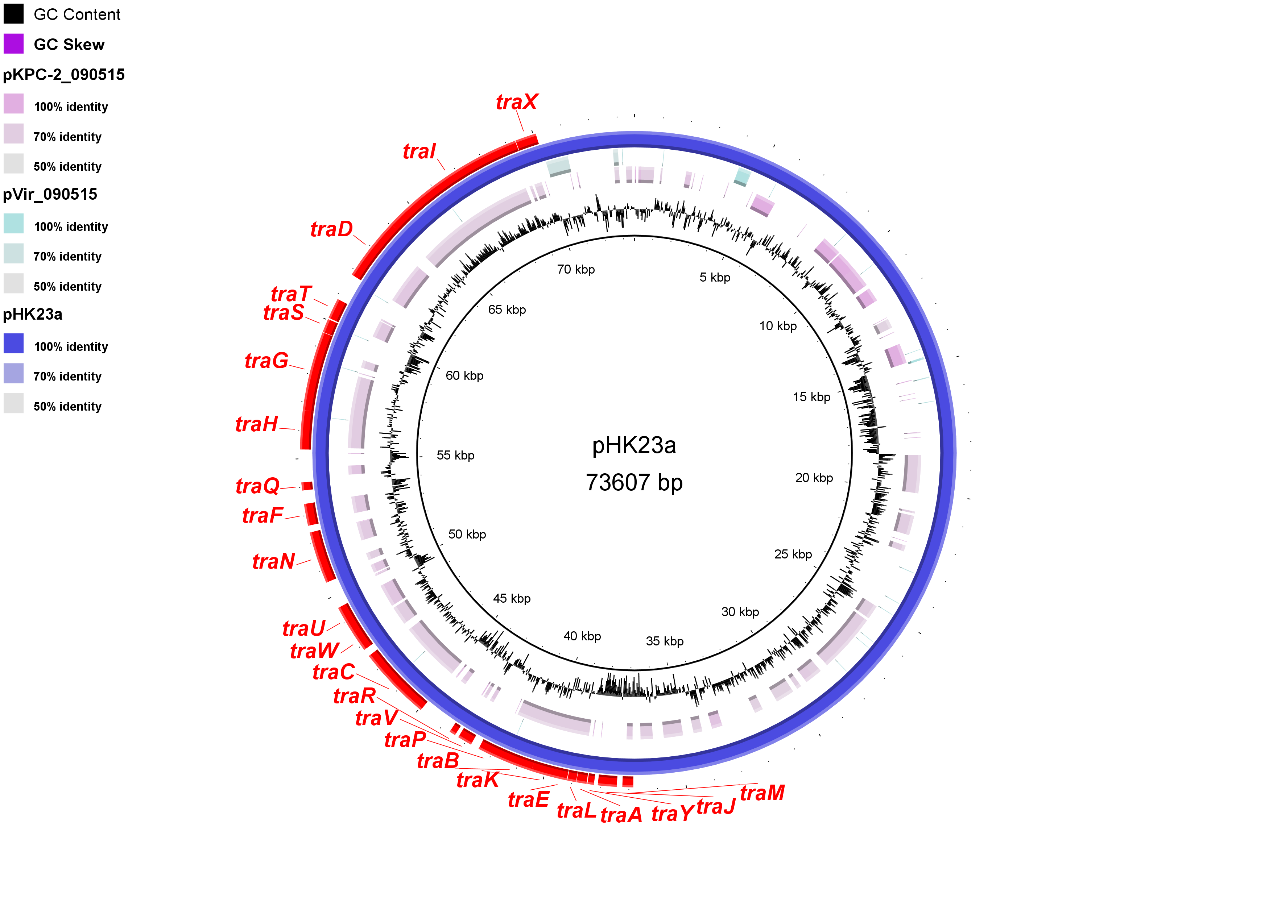


**Fig. S4. Alignment of pKPC-2_Vir with pKPC-2_090515 and pVir-2_090515.**

pKPC-2_090515 and pVir_090515 had a 12-bp identical region (shown in dark blue), while pKPC-2_Vir had two such 12-bp regions. Genes encoding antimicrobial resistance, virulence, plasmid replicons, and conjugation are shown.


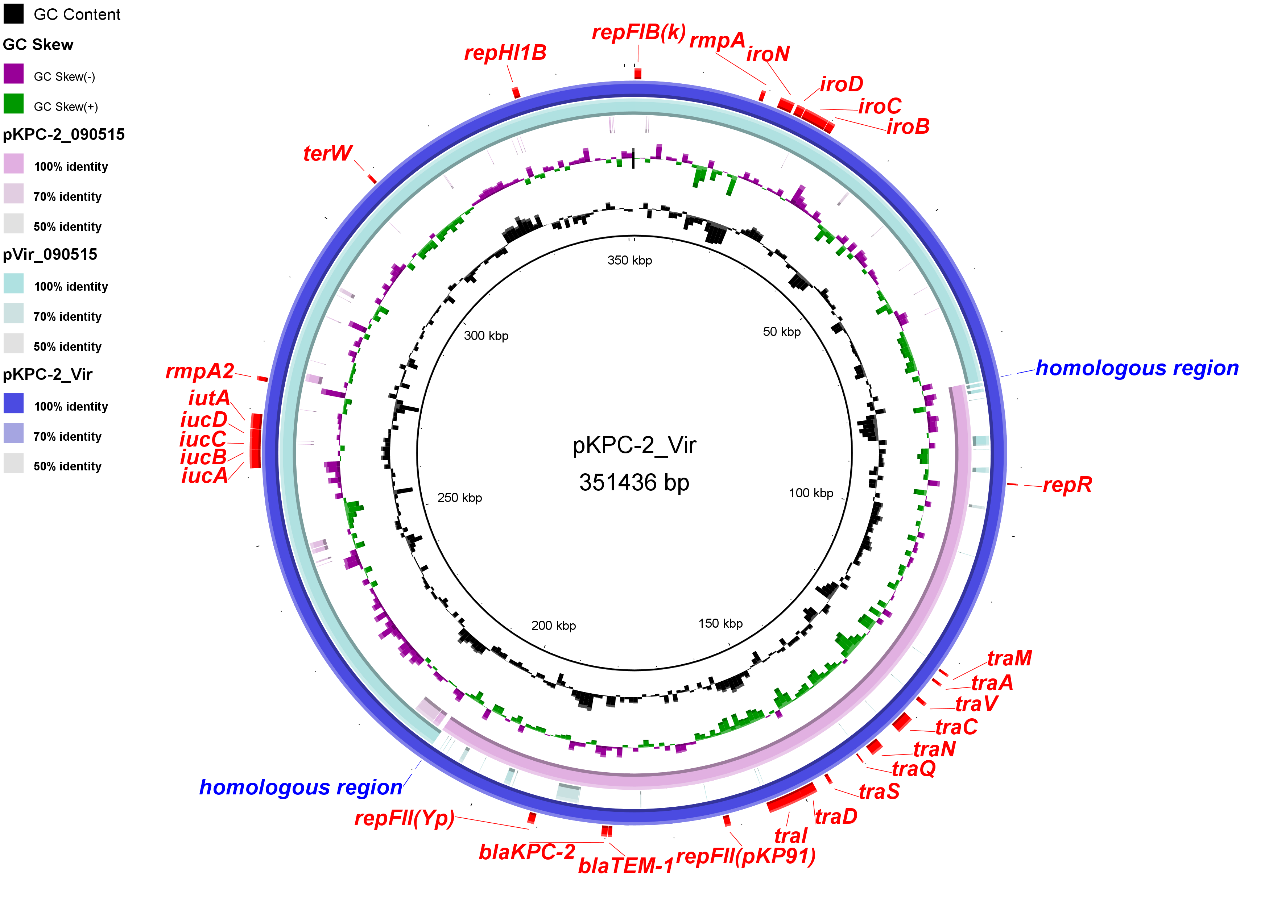


**Fig. S5.** The gel electrophoresis results of the PCR for fusion sites. In conjugation, strain 090515 was the donor, strains J53 and 115112 were the recipients, and Tx090515Vir and Tx11511 were the corresponding transconjugants containing the hybrid plasmid pKPC-2_Vir. PCR for fusion sites was negative for the donor and recipients but was positive for the two transconjugants. PCR primers are listed in Table 1.

**
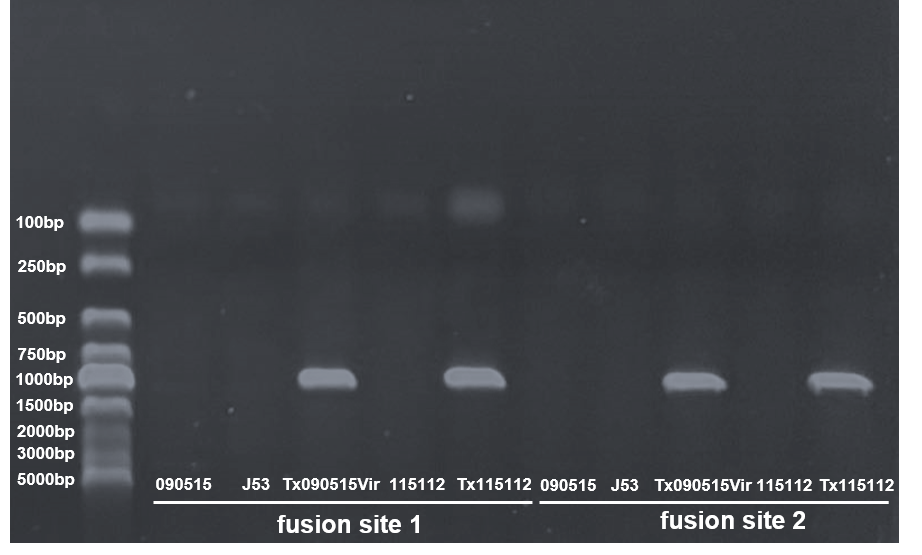
**

Table S1. Survival rate of *Galleria mellonella* in the virulence assay

| CFU | 090515 | 090249 | 090276 | 090267 | J53 AziR | Tx090515Vir | Tx115112 | 115112 | PBS |
| --- | --- | --- | --- | --- | --- | --- | --- | --- | --- |
| 12 h |  |  |  |  |  |  |  |  |  |
| 10^5^ | 0.46±0.26* | 0.81±0.06 | 0.31±0.12* | 0.92±0.09 | 0.93±0.11 | 0.94±0.06 | 0.67±0.22 | 0.69±0.35 | 0.98±0.02 |
| 10^6^ | 0.19±0.22* | 0.33±0.13* | 0.18±0.10* | 0.85±0.09 | 0.85±0.09 | 0.81±0.06 | 0.48±0.19 | 0.63±0.38 |  |
| 10^7^ | 0.02±0.04* | 0.16±0.13* | 0.06±0.06* | 0.75±0.06 | 0.75±0.13 | 0.71±0.07 | 0.29±0.28 | 0.52±0.28 |  |
| 24 h |  |  |  |  |  |  |  |  |  |
| 10^5^ | 0.31±0.31* | 0.54±0.22 | 0.21±0.09* | 0.89±0.07 | 0.79±0.16 | 0.85±0.20 | 0.60±0.19 | 0.63±0.38 | 0.98±0.02 |
| 10^6^ | 0.14±0.08* | 0.10±0.09* | 0.11±0.06* | 0.79±0.14 | 0.69±0.17 | 0.71±0.13 | 0.44±0.19 | 0.56±0.39 |  |
| 10^7^ | 0* | 0.06±0.06* | 0.04±0.03* | 0.71±0.13 | 0.52±0.07 | 0.63±0.11 | 0.25±0.25 | 0.46±0.32 |  |

J53 AziR was the azide-resistant variants of *E. coli* J53; 090515 (the strain in this study), 090276 and 090249 were ST592 K57, ST11 K64 and ST23 K1 carbapenem-resistant hypervirulent *K. pneumoniae*, respectively; 115112 and 090267 were the ST11 K64 carbapenem-resistant non-hypervirulent *K. pneumoniae*; Tx090515Vir and Tx115112 were the transconjugants containing pKPC-2_Vir of J53 AziR and115112, respectively.

Percentages (%, mean ± standard deviation) of larvae (n=16 for each strain) survived after infected with the corresponding strain at an inoculum of 10^5^ to 10^7^ CFU/ml at different time points (h) are shown. The experiments were performed in triplicate. An unpaired two-sided Student’s *t-*test was performed to analyze the statistical difference using SPSS 26.0.

*The survival rate of *Galleria mellonella* infected with hypervirulent strain 090515, 090249 and 090276 was significantly different from those infected with non-hypervirulent strain 090267.
